# Evaluation of Monkeypox as an Unrecognized Sexually Transmitted Disease: A Rare Case of Monkeypox Infection with HIV and Syphilis Coinfection

**DOI:** 10.7759/cureus.42326

**Published:** 2023-07-23

**Authors:** Ahmed Ali Aziz, Maleeha Saleem, Sara L Wallach, Shazia M Shah

**Affiliations:** 1 Internal Medicine, Capital Health Regional Medical Center, Trenton, USA; 2 Internal Medicine, Hackensack Meridian School of Medicine, Seton Hall University, Nutley, USA

**Keywords:** sexually transmitted infection, infectious disease transmission, syphilis rash, hiv aids, monkey pox

## Abstract

Monkeypox (MPX) virus is endemic in Africa. However, since May 2022, many cases have been reported worldwide in many non-endemic regions as well. The virus usually spreads from animals to humans or from humans to humans through respiratory droplets or after contact with infected lesions. In the recent outbreak of MPX, many cases did not have any travel history to endemic areas and were reported in men who have sex with men (MSM) along with the diagnosis of other sexually transmitted diseases (STDs). However, MPX is not yet considered a sexually transmitted infection (STI), even though a relationship between MPX and other STIs may exist with a possible facilitating action on their spreading. We present a similar case of MPX infection in an MSM patient with concomitant HIV and syphilis infections and no travel history to an endemic area.

## Introduction

Monkeypox (MPX) virus is an orthopoxvirus [1]. The first human case of MPX was reported in 1970 in the Democratic Republic of Congo and, since then, it has been endemic in Africa, where sporadic outbreaks of MPX have been reported. However, since early May 2022, many cases were detected in over 50 countries worldwide prompting the World Health Organization (WHO) to declare MPX outbreak as a public health emergency of international concern on July 23, 2022. MPX cases in the current outbreak differed in clinical characteristics from those previously reported. MPX infection was detected disproportionally higher in bisexuals and men who have sex with men (MSM) with co-existing sexually transmitted infections (STIs) raising concerns about potential sexual transmission of MPX [2]. MPX is a zoonotic disease, not historically associated with sexual contact; however, contact with skin lesions during sexual activity might be a mode of transmission [3]. Patients usually present with fever and myalgias followed by the development of a vesicular rash that spreads in a centrifugal direction. Many STIs present with a similar prodrome and rash and, hence, the diagnosis of MPX can be difficult. Severe cases of disseminated MPX disease have been reported in immunocompromised patients such as those with HIV infection. Here, we present a patient who presented with a disseminated rash and tested positive for MPX and concomitant HIV and syphilis infections.

## Case presentation

A 36-year-old male patient with no past medical history presented to our hospital for evaluation of a painful and itchy rash that started on his face and then spread peripherally to his trunk, arms, and legs. Two days prior, he was seen at the emergency department of a nearby hospital for fever, vomiting, abdominal pain, and myalgias and was discharged on symptomatic treatment. He reported having unprotected sexual intercourse with multiple male partners and mentioned that one of his sexual partners tested positive for syphilis and did not recall any other diseases in any of his other partners. He denied any recent travel. On admission, his vitals were stable and within normal limits. On physical examination, he had 5 to 10 mm diffuse, tender, pustular lesions with surrounding erythema over his face, trunk, arms, and legs, accompanied by bilateral tender cervical, axillary, and inguinal lymphadenopathy. Due to several MPX cases reported worldwide since January 2022 with a similar presentation of a vesicular to pustular rash in MSM, the patient was admitted to the hospital and managed with contact and airborne isolation precautions until MPX and Varicella Zoster Virus (VZV) infections were ruled out. Routine blood tests including serum biochemistry, liver function tests, and complete blood count were all within normal limits (Table 1). Due to his sexual history, he was also tested for sexually transmitted diseases (STDs). His HIV serology came back positive. Syphilis serology was also positive and showed a rapid plasma reagin (RPR) titer of 8. Test results for VZV, hepatitis B virus (HBV), hepatitis C virus (HCV), Chlamydia trachomatis and Neisseria gonorrhea were negative. Swabs taken from deroofed skin lesions from two different parts of the body (trunk and legs) returned positive for MPX virus (Table 2). The Department of Health was notified about the MPX patient, and infectious disease was consulted for their expertise in the management of MPX infection with concomitant HIV and syphilis infections. In consultation with infectious disease patient was started on tecovirimat for treatment of MPX infection, anti-retroviral therapy (ART) for HIV infection and penicillin for syphilis infection. Oxycodone-acetaminophen was prescribed for pain control. His rash and pain improved, and he was discharged on day 7 with instructions to continue contact and airborne isolation at home until all the lesions became scabs and resolved. He was scheduled a telemedicine follow-up appointment one week and an HIV clinic appointment one month after discharge. At his follow-up telemedicine appointment, he reported his rash and pain had greatly improved. 

**Table 1:** Blood test results at the time of admission

Lab Parameters (units)	Reference Range	Result
Sodium (mmol/L)	135-145	136
Potassium (mmol/L)	3.5-5.5	4
Chloride (mmol/L)	96-106	101
Blood Urea Nitrogen (mg/dL)	Jun-24	15
Creatinine (mg/dL)	0.7-1.3	0.8
Bicarbonate (mmol/L)	22-29	24
Glucose (mg/dL)	70-100	97
Aspartate Aminotransferase (U/L)	13-39	14
Alanine Aminotransferase (U/L)	Jul-52	10
Alkaline Phosphatase (U/L)	34-104	52
Lactate (mmol/L)	0.5-2.0	1.2
White Blood Cells (k/uL)	3.8-10.2	8.4
Hemoglobin (g/dL)	12.9-16.7	13.1
Hematocrit (%)	39.2-48.8	39.3
Platelets (k/uL)	150-450	258

**Table 2:** Serology results of various infectious diseases tested RNA: Ribonucleic Acid, PCR: Polymerase Chain Reaction, DNA: Deoxyribonucleic Acid, SARS-Cov-2: Severe Acute Respiratory Syndrome Coronavirus 2; VZV: Varicella Zoster Virus; IgG: Immunoglobulin G; IgM: Immunoglobulin M; RPR: Rapid plasma reagin; MPX: Monkeypox

Infectious Disease Serology (units)	Reference Range	Result
HIV 1 and 2 Antibody with P24 Antigen	Non-reactive	Reactive
HIV 1 Antibody	Non-reactive	Reactive
HIV 2 Antibody	Non-reactive	Non-reactive
HIV 1 RNA PCR Quantitative – Log 10 (Log 10 Copies /mL)	< 1.60	5.20
HIV 1 RNA Quantitative (Copies /mL)	< 40	105187
Hepatitis B Surface Antigen	Non-reactive	Non-reactive
Hepatitis C Antibody	Non-reactive	Non-reactive
RPR	Non-reactive	Reactive
RPR Titer (1:NN)	Non-reactive	1 : 8
Treponema Pallidum Antibody	Non-reactive	Reactive
VZV DNA Qualitative PCR	Not detected	Not detected
VZV IgG Antibody (Index)	>= 165.00	1273.00
VZV IgM Antibody (Index)	<= 0.90	0.84
Chlamydia trachomatis DNA Urine	Not detected	Not detected
Neisseria gonorrhea DNA Urine	Not detected	Not detected
Respiratory PCR Panel SARS-Cov-2	Not detected	Not detected
MPX PCR	Not detected	Detected

## Discussion

MPX is not historically associated with sexual contact. However, close contact with infected skin lesions during sexual activity might facilitate transmission [4,5]. Patients typically present with non-specific symptoms of fever, chills and myalgias followed by the development of a vesicular rash one to four days later that spreads in a centrifugal distribution [4,6]. The rash usually begins as macules that later progress to vesicles, pustules, and scabs [7]. Patients are infectious from the time of onset of symptoms until all the lesions become scabs [8].

In the current MPX outbreak, MPX infection has been reported after sexual contact with infected persons, especially in MSM [1]. It might be possible that MPX is an unrecognized STI. Our patient was an MSM and presented with a concomitant acquisition of HIV, syphilis and MPX infections after sexual contact with male partners. Thornhill et al. reported the presence of STIs (gonorrhea, chlamydia, or syphilis) in 109 of the 377 persons (29%) who had MPX infection and underwent testing for STIs [9]. Potential sexual transmission of MPX is also supported by studies that mentioned MPX lesions at the site of initial sexual contact such as penile MPX lesions prior to the onset of systemic illness [10]. Kristine et al. reported in their case report study in 2022 that the most common anatomical sites for MPX rash were the anogenital area [11]. However, in order to recognize MPX as a possible STI, further large-scale, multi-center studies will be required.

Cutaneous manifestations of MPX are very similar to other infections such as HSV, syphilis, early-stage measles, chickenpox, molluscum contagiosum, and rickettsial diseases [12]. MPX should, hence, be considered in the differentials of a vesicular or pustular rash, and patients should be kept in contact and airborne isolation until MPX is ruled out. We recommend that patients suspected to have a MPX rash should be hospitalized for isolation, diagnostic evaluation and testing for any co-existing STIs as we did for our patient.

On our review of literature we found many studies reporting patients with MPX having concomitant HIV infection. Mungmunpuntipantip et al. found 27 cases of concurrent MPX and HIV infection in their systematic literature search of PubMed articles [13]. In a Morbidity and Mortality Weekly Report (MMWR) report published in September 2022, 1,969 cases of MPX were analyzed and HIV prevalence was 34% [14]. Thornhill et al. reported MSM with HIV had a higher prevalence in the population infected with MPX in the current outbreak [9]. Hence, we recommend MPX patients should be screened for HIV. Patients with MPX and co-existing HIV infection have been reported to have poorer clinical outcomes related to MPX infection. Complications, such as pneumonitis, encephalitis, or bacterial superinfection, can occur in immunocompromised patients [12]. Prompt diagnosis and treatment of both MPX and HIV should be initiated to prevent complications as we did for our patient. As per Centers for Disease Control and Prevention (CDC) 2022 guidelines, tecovirimat is the current drug of choice indicated for patients with severe MPX and for patients at high risk of severe disease like immunocompromised patients living with HIV. ART for HIV infection must be initiated to prevent the risk of developing bacterial superinfection [9]. There is no specific vaccine for MPX. However, smallpox vaccine is currently being used as post-exposure prophylaxis in patients with close contact with MPX [12]. There are two United States Food and Drug Administration (FDA)-approved vaccines, JYNNEOSTM and ACAM2000®, for the prevention of smallpox, and they have been shown to be effective against MPX as well [11]. Mild cases of MPX infection may be self-limiting and require only routine care and isolation without the need for antiviral drugs [15]. 

## Conclusions

Our case is different from other reports, in that the MPX infection presented with multiple concomitant STIs that resulted in a disseminated disease presentation. Our case report highlights several important points. First, MPX should be considered a differential in patients who present with a vesicular or pustular rash, and patients should be kept in contact and airborne isolation until MPX is ruled out. Second, MPX might be sexually transmitted and all such patients should be tested for any co-existing STIs. Third, many patients with MPX have been reported to have HIV as well. HIV might affect the clinical presentation and clinical course of the disease. Patients with MPX and HIV should be immediately treated with tecovirimat and ART to prevent bacterial superinfections and complications. We advocate aggressive management of MPX infection in immunocompromised patients living with HIV.
